# Flexibilization of Fasting for Laboratory Determination of the Lipid Profile in Brazil: Science or Convenience?

**DOI:** 10.5935/abc.20180174

**Published:** 2018-11

**Authors:** Caio Maurício Mendes de Cordova, Caroline Galgowski

**Affiliations:** Fundação Universidade Regional de Blumenau (FURB), Blumenau, SC - Brazil

**Keywords:** Fasting, Cholesterol, Lipids, Triglycerides, Cholesterol, LDL, Cholesterol, HDL

A statement endorsed by medical specialty associations has been published in our country recommending the flexibilization of fasting before blood drawing for the laboratory determination of the lipid profile encompassing total cholesterol (TC), high-density lipoprotein cholesterol (HDL-c), triglycerides (TG) content and the corresponding calculation of non-HDL-cholesterol (TC - HDL-c).^1^ It was considered that non-fasting results do not clinically differ from fasting ones, and prospective studies and meta-analyses have consistently demonstrated that non-HDL-C at a non-fasting state would be at least as good as LDL-c in the prediction of CVD. It was also recommended that when TG > 4.52 mmol/L the formula proposed by Martin et al.^2^ should be used for LDL-c estimation.

The statement was based on the European Consensus on the matter published by Nordestgaard et al.^3^ However, the automatic application of this approach in Brazil deserves deeper consideration, considering the impact that it may cause on patient care. Furthermore, it is far from a consensus among clinical laboratory scientists and professionals in the country, as it became evident during the 44^th^ Brazilian Congress of Clinical Analysis held last June 11-14^th^, 2017, and the 51^st^ Brazilian Congress of Clinical Pathology and Laboratory Medicine, held last September 26-29^th^, 2017.

Indeed, a non-fasting non-HDL-c result would be at least equivalent to LDL-c for goal setting.^4^ However, a non-fasting LDL-cholesterol, as well as non-fasting non-HDL-c, could be less sensitive for CVD prediction,^5^ especially in women.^6^ This possible issue ought to be evaluated judiciously and independently in our specific population.

Secondly, it should be noted that the treatment target for non-HDL-c is simply 0.8 mmol/L (30 mg/dL) higher than the respective target for LDL-c.^7^ This was set in an empirical manner, considering an average value of 0.8 mmol/L for VLDL-c. Obviously, this is not consistent with reality, especially in a post-prandial state. On the other hand, the treatment target levels for LDL-c are well established, based on large prospective studies for decades of sound scientific work.

Third, the main motives for a non-fasting blood draw as suggested by the European consensus^3^ and the Brazilian statement^1^ seem to be more commercially driven than scientifically. The rationale included an alleged “inconvenience by having to return on a separate visit for a fasting lipid profile…, a laboratory burden due to a large volume of patients coming for tests in the morning…, a burden for clinicians to review and make decisions based on the findings of the lipid profile at a later date…”, and a hypothesized improved “patient compliance with lipid testing”.

Only the last motivation may have some scientific background but it yet remains to be proved. It also should be noted that blood sample drawing procedures in Brazil are quite different from those practiced in Western Europe and in the USA. In those countries, biological samples are often drawn right after the consultation with the clinician, at the clinic or hospital; the samples are collected at scheduled times by the laboratory logistics and the result is directly reported to the physician. The patients do not even know what a clinical laboratory is; they just know that their blood samples go somewhere to be analyzed by people who they have no idea what their skills and background are. In Brazil, by law, the laboratory results belong to the patients, and non-hospitalized patients often come to the laboratory collection facility, unless a home visit is scheduled, for blood drawing or other biological sample collection days after the first consultation, where they receive adequate instructions regarding the pre-analytical requirements for each requested test. The realities are completely different.

Fourth, precisely derived from the point above, the impact of these recommendations have not yet been evaluated on the patient's behavior regarding the required fasting for *other* laboratory tests. And even worse, we have already observed movements by some corporations indicating that fasting for *any* laboratory test would be no longer necessary. From the technical and scientific point of view, non-fasting blood samples are not suitable for measurement of several analytes that are influenced by meals, such as blood cell counts, hemoglobin, albumin, bilirubin, phosphate, calcium, magnesium, potassium,^8^ insulin, growth hormone, glucagon, chloride, urine pH, and also those affected by diurnal variation, such as ACTH, catecholamines, TSH, PTH, renin, aldosterone, ALT, AST, alkaline phosphatase, blood urea nitrogen and iron,^9^ to name a few. As it has been said,^10^ in clinical laboratory medicine, no sample would be preferred to a bad sample, if one wishes to attain rigorous standards when providing clinicians with reliable laboratory information. The overall impact of the proposed non-fasting blood sample draw on the eventual rejection of the patient’s samples has yet to be determined, due to the presence of other requested laboratory tests that need fasting and/or morning draw.

And fifth, finally, the suggested Martin’s formula still uses TG in its calculations, a parameter that has been demonstrated by many authors not to be correlated with LDL-c or TC. Martin et al.^2^ have made a huge mathematical effort to achieve a satisfactory result to include TG in the calculation. And most importantly, this equation has to be validated or at least evaluated, in other populations before being universally recommended. For instance, the proposed Martin’s formula, as well as ours, was evaluated in comparison to newly proposed formulas for LDL-c estimation in Iran, and the former was demonstrated to not add value to the estimations in a small cohort.^11^

Anyway, LDL-c remains a frequent parameter requested at clinical laboratories in medical routine, and will likely continue to be so, hence precise methods for its estimation are needed when its direct measurement is not available. A simple and accurate equation developed and evaluated in the Brazilian population has already been developed.^12^ It should be noted that this equation performs equally well, for instance, in populations from Germany and United Kingdom,^13^ but not as well in others, such as in South Africa,^14^ Spain,^15^ and Thailand.^16^ It seems that the debate on which method to use for LDL-c determination, in each particular population of the globe, is more open than defined.^17^

Sadly, history is full of examples demonstrating that when corporate interests meet with poor science, the only losers are science itself, and patient care. It is apparent and worthy of concern that the Brazilian 'consensus' has recommended the use of an equation for LDL-c estimation that was not validated in the local population and was moved by reasons that are driven more by convenience than by rigorous and unbiased science.
